# Efficient Multiple Sequences Alignment Algorithm Generation *via* Components Assembly Under PAR Framework

**DOI:** 10.3389/fgene.2020.628175

**Published:** 2021-02-04

**Authors:** Haipeng Shi, Haihe Shi, Shenghua Xu

**Affiliations:** ^1^School of Information Management, Jiangxi University of Finance and Economics, Nanchang, China; ^2^School of Software, Jiangxi Normal University, Nanchang, China; ^3^School of Computer and Information Engineering, Jiangxi Normal University, Nanchang, China

**Keywords:** multiple sequence alignment algorithm, domain component, algorithm generation, convenient software development platform, bioinformatics

## Abstract

As a key algorithm in bioinformatics, sequence alignment algorithm is widely used in sequence similarity analysis and genome sequence database search. Existing research focuses mainly on the specific steps of the algorithm or is for specific problems, lack of high-level abstract domain algorithm framework. Multiple sequence alignment algorithms are more complex, redundant, and difficult to understand, and it is not easy for users to select the appropriate algorithm; some computing errors may occur. Based on our constructed pairwise sequence alignment algorithm component library and the convenient software platform PAR, a few expansion domain components are developed for multiple sequence alignment application domain, and specific multiple sequence alignment algorithm can be designed, and its corresponding program, i.e., C++/Java/Python program, can be generated efficiently and thus enables the improvement of the development efficiency of complex algorithms, as well as accuracy of sequence alignment calculation. A star alignment algorithm is designed and generated to demonstrate the development process.

## Introduction

Alignment is a common and important approach in biology study. In the research of bioinformatics (Wang et al., 2015), biological sequence alignment is one of the important processes of similarity analysis between unknown and known molecular sequences, the basis of biological sequence analysis and database search, and used in the sequence assembly. It is the key link to apply high-performance computing to biology.

Sequence alignment is a technique for identifying regions of sequence similarity by arranging genome sequences to obtain the function, structure, or evolutionary relationship between the sequences to be aligned. With the implementation of the Human Genome Project, the development of sequencing technology has produced a large amount of raw sequence data about biological molecules. For example, Illumina HiSeqX Ten can generate approximately 3 billion 2 × 150 bp paired-end sequencing data within 3 days (Illumina, 2016). Challenged with such a wealth of genome sequence data, to efficiently process and analyze these data, to compare similar regions and conserved sites between the two sequences, to seek sequence homology structures, and to reveal biological heredity, variation, and evolution, etc., have become the main motivations for the research of sequence alignment algorithms.

At present, most of the research on alignment algorithms focus on specific problems (Isa et al., 2014; Cattaneo et al., 2015; Chattopadhyay et al., 2015; Huo et al., 2016) or specific algorithm optimization (Farrar, 2007; Houtgast et al., 2017; Junid et al., 2017) in the field of sequence similarity analysis, but less on the whole problem domain, so it is difficult to get an algorithm component library with a higher level of abstraction and suitable for the whole field of sequence similarity analysis. To some extent, this leads to the redundancy of the sequence alignment algorithm and the errors that may be caused by the artificial selection algorithm. It also makes it difficult for people to effectively understand the algorithm structure and ensure the correct use of the algorithm, which reduces the accuracy of the sequence similarity analysis. Because of the specificity and low-level abstraction of existing algorithms, researchers need to spend a lot of time to learn and use such algorithms, and it is also difficult to locate and solve the errors generated by the algorithms; thus, maintainability and reusability of the algorithms are reduced, and the burden of sequence similarity analysis is increased.

Sequence alignment algorithms can be divided into pairwise alignment algorithms and multiplesequence alignment algorithms (Zhan et al., 2019, 2020). Among them, the most classic solution to the pairwise sequence alignment algorithm is dynamic programming. We studied the field of dynamic programming–based pairwise sequence alignment algorithm (DPPSAA) in the early stage and established a domain component library (Shi and Zhou, 2019), which has been successfully applied to the problem of pairwise sequence alignment algorithm. However, the multisequence alignment algorithm is rather complex. Because of its non-deterministic polynomial (NP)-complete (Wang and Jiang, 1994), current researches are all devoted to finding the optimal approximate solution. With the increase of the complexity and difficulty of the multisequence alignment algorithm, the reliability and efficiency of the algorithm are difficult to be guaranteed.

Based on the previous work, this article adopts the formal method PAR (Xue, 1997, 2016; Shi and Xue, 2009, 2012; Xue et al., 2018) to describe, construct, transform, and refine the components, models, and frameworks related to the multisequence alignment algorithm and expand PAR platform to support the generation of effective multiple sequence alignment algorithm *via* component assembly. The multilevel different models in the algorithm development process are unified under the PAR framework to effectively ensure the reliability of the resulting algorithm and improve the efficiency of algorithm development.

Through in-depth analysis of the field of multiple sequence alignment algorithms, based on the component library of the DPPSAA domain, some algorithm components have been improved and added, and a component library of multiple sequence alignment algorithms on top of the component library of the DPPSAA domain was established. Finally, an example, the successful assembly of the star alignment algorithm and the automatic generation of the C++ program, is shown.

## Algorithm Generation Under the Par Framework

### Related Work

On the basis of the component library in the DPPSAA domain, this article has carried out the research on the algorithm design and program generation of multiple sequence alignment algorithms under the PAR framework.

#### PAR

The PAR framework includes two parts: software formal method and convenient software development platform. The PAR method is composed of a generic algorithm design language Radl, a generic abstract programming language Apla, systematic methodology for algorithms and programming. It combines two high-efficiency techniques, i.e., partition and recursion used in special problems, covering a variety of known algorithm design techniques such as dynamic programming, greedy, divide and conquer, and so on. It can be used as a unified method of algorithm generation to avoid the difficulty of making choices among the existing algorithm design methods. The PAR platform is composed of Apla to C++/C#/Java/Python program generation systems and realizes the automatic generation of algorithmic programs such as sequential programs, parallel/concurrent programs, and database applications.

Practice has proven that the productivity of complex algorithm program and database application software can be greatly improved by using the language, method, series algorithm, and program automatic generation tool provided by PAR. Many military departments, such as the National General Equipment Department, Beijing Military Region, and armored academy, have taken the lead in applying these achievements to the construction of China’s important military projects and have achieved remarkable military and economic benefits. The PAR framework has been appraised by the expert group of the Ministry of Science and Technology of China as “having the international advanced level, among which the theoretical framework of the correctness of the complex algorithm program has the international leading level.”

#### DPPSAA Domain Model and Component Library

In Shi and Zhou (2019), we analyzed the characteristics of DPPSAA, extracted the common and variable features and the constraints and dependencies between them, established the DPPSAA domain model and its algorithm component interaction model, and further implemented the models using the abstract programming language Apla to form a highly abstract DPPSAA component library, in order to automatically or semiautomatically assemble components to generate sequence alignment algorithms for specific fields, thereby reducing the error rate and time cost of manual selection algorithms for sequence similarity analysis, improving the efficiency of algorithm execution, and even assembling a more efficient new sequence alignment algorithm based on dynamic programming.

The experimental results show that the DPPSAA algorithm component library has a certain degree of practicability and has good expected results. It can be seen from the domain realization process that the domain feature model is a formal description at a higher level of abstraction, which not only makes the specific composition characteristics and dependencies of the algorithm clearly displayed, but also is very helpful for understanding the overall architecture of the algorithm. Moreover, the establishment of the feature interaction model makes it easier to specify the specific configuration knowledge required by the algorithm in the domain implementation process and then automatically assemble the components in the DPPSAA algorithm component library to design the desired algorithm, without paying too much attention to the details of algorithm implementation.

### Algorithm Generation Process

Based on a large amount of practical work carried out in the early stage, combined with related methodologies such as PAR and domain engineering, the development of multisequence alignment algorithms can be divided into two parts: reuse-oriented development and application reuse development.

For reuse-oriented development, it can be divided into the following steps:

1. Analyze the algorithm family in the field of multiple sequence alignment, and establish the domain model.
2. Formally describe the component function specifications.
3. Use the PAR method to design abstract Apla algorithm components, use the PAR platform to obtain highly reliable executable language-level components, and expand the PAR platform component library in a self-expanding manner.

The process of designing a specific problem-solving algorithm and generating a program is a development process of application reuse:

1. Analyze and (formally) characterize the specific problem to be solved.
2. Determine the algorithm components required for assembly.
3. The Apla abstract language is used to describe the assembly process, and the executable program corresponding to the specific algorithm is automatically generated through the PAR platform.

The introduction of the PAR framework reduces the operational difficulty of algorithm component assembly and improves the automation of algorithm component assembly.

## Star Alignment Algorithm

### Algorithm Idea

The star alignment algorithm (Zou et al., 2009, 2015) is a heuristic fast approximation algorithm for typical multisequence alignment. It compares all sequences in pairs and selects the sequence with the highest alignment score with other sequences as the central sequence. Then, continue to compare with other sequences to obtain the final alignment result. When adding subsequent sequences to the alignment process, follow the “leave blank once, leave blank everywhere” rule, which cannot guarantee the ultimate result of the alignment.

For example, for the sequence s1 = CGCT, s2 = GCGT, s3 = CCTG, the pairwise alignment results of the sequences s1, s2, and s3 are shown in Table 1.

**Table 1:** Distance matrix of s1, s2, and s3.

	s1	s2	s3	Score
s1		−1	−1	−2
s2	−1		−2	−3
s3	−1	−2		−3

**Figure 1:**
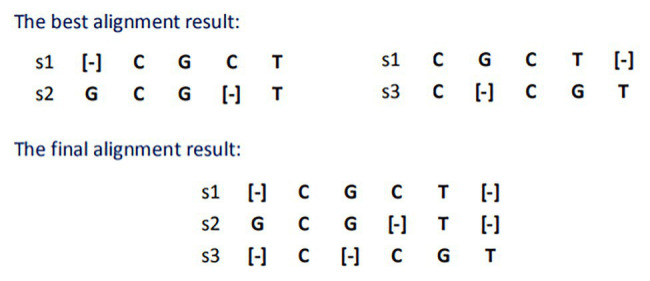
Result of star sequence alignment.

The star alignment algorithm adds the alignment scores of each sequence to other sequences and selects the sequence with the largest score as the central sequence. Therefore, in this case, s1 is selected as the center sequence, and the best alignment result and the final merge result with S2 and S3 are shown in Figure 1.

### Algorithm Component and Apla Implementation

Using feature modeling knowledge and performing process analysis on star alignment algorithms, we will know that multisequence alignment is mainly used as the core service of star alignment algorithms in the star alignment process. The multiple sequence alignment service is mainly based on the pairwise sequence alignment, by selecting the optimal pairwise sequence alignment result as the central sequence, and then continuously adding the suboptimal sequence to the alignment until the final multisequence alignment result is obtained. After analyzing the execution process of the star alignment algorithm, the multisequence alignment operation service mainly consists of the following features (the component name of the corresponding feature in parentheses): sequence legality check (msa_check), distance matrix (dist_Matrix), pairwise alignment manipulation (align_manipulation), center sequence selection (msa_center), remember alignment space (rmb_space), multisequence alignment result output (msa_op_result), and so on. Among them, sequence legality check, pairwise alignment manipulation, distance matrix, and center sequence selection are mandatory features in the star alignment algorithm, and the multisequence alignment result output feature mainly depends on the remember alignment space feature; that is, when the assembly algorithm contains a multisequence alignment result output component, it will include and implement the remember alignment space component by default.

Taking DPPSAA as the basis of sequence alignment, generic programming language Apla is used to abstractly represent the star alignment algorithm, which can realize star alignment algorithm by standardized assembly. Here, we expand on the basis of the component library in the DPPSAA domain, so that the component library in this domain can be used to assemble and implement the star alignment algorithm. We perform Apla representation of the extended component as follows:

1. Sequence legality check
  msa_check is an extension based on the check component in the DPPSAA field that can be used to detect multiple sequences. The Apla process statement is:
  procedure msa_check(String str[]);
  where str[ ] represents the base string array for multiple sequence alignment.
2. Distance matrix
  dist_Matrix means that all pairwise alignment scores participating in multisequence alignments are returned as distance matrix elements, and the component uses pairwise sequence alignment operations as its generic parameters. The prototype of the Apla function is as follows:
  function dist_Matrix (procalign_manipulation(sometype elemMatrix; ADT dp_mode(eM:elemMatrix); op_mode (func score_op():integer; proc traceback (proc print_align(); proc print_extrude() =NULL)); result:boolean; eM: elemMatrix; s:String; t:String))):integer[ ][ ].
3. Center sequence selection
  The msa_center component is an important part of the components library of multiple sequence alignment algorithm. This component can be used to select the best alignment in all pairwise alignments; take the best alignment sequence in the alignment as the center sequence, and then iteratively add the remaining sequences to obtain the best multiple sequence alignment results. The function prototype is as follows:
  function msa_center(dist[][]: integer):integer;
  The array dist represents the array returned by the distance matrix, and the component returns the index value of the center sequence.
4. Remember alignment space
  In the star alignment algorithm, the algorithm follows the rule of “leave blank once, leave blank everywhere” when adding subsequent sequences to the alignment process. Therefore, the role of the rmb_space component is to remember the space inserted during each sequence alignment. The function prototype is as follows:
  function rmb_space(): integer[][];
5. Multisequence alignment result output
  This component inserts the space index value obtained in (4) into the sequence to output the final multisequence alignment result. This component can be implemented with the following Apla process:
  procedure msa_op_result(space[][]:integer);

### Star Alignment Algorithm Generation

Using the Apla-C++ conversion system, the aforementioned component library is converted into the corresponding C++ component through the combination of automatic conversion and manual conversion, which can be used to generate the star alignment algorithm program and conduct test analysis to obtain experimental results. This section shows only the three main components: dist_Matrix component, msa_center component, and rmb_space component.

As the star alignment algorithm requires the pairwise sequence alignment manipulation, and the alignment score result value is used as the element of the distance matrix, the dist_ matrix component needs to use the sequence alignment manipulation in DPPSAA as its generic parameter to obtain the score value of the pairwise alignment of all sequences. In the process of converting the Apla program to the C++ program, it is first necessary to assemble the components in DPPSAA to form a pairwise sequence alignment algorithm and design the pairwise sequence alignment algorithm as an independent function as the function pointer parameter of the distance matrix component, which reduces the dependency between the pairwise sequence alignment algorithm and the distance matrix. Here, we set the pairwise sequence alignment algorithm to NW algorithm and return the pairwise sequence alignment scores. The C++ code is as follows:

class MsaNW{//NW algorithm assembly

public:

int Msa_NW(Score_matrix_mani& matrix,const std::string& s,const std::string& t){

matrix.apply_memory();

matrix.Memory_Score_of_Matrix(&Init_Score_matrix::Init_Score_matrix1, matrix.get_Matrix(), matrix.getPenaltyMatrix(), matrix.get_length_s(), matrix.get_length_t());

dp_mode dp_NW;

dp_NW.align_and_score(matrix,&set_and_remember::set_and_remember1);

return matrix.the_Last_element_score();

}

}

The C++ program obtained by transforming the dis_matrix component is as follows:

class dist_Matrix{

int** dist; //distance matrix

int* row_sum;//sum of row

int seqs_num;//number of sequences

public:

void Dist_Matrix(int(MsaNW::*Msa_NW)(Score_matrix_mani&, const std::string&, const std::string&),std:string* seqs, Score_matrix_mani** matrix)//final score {...}

void sum_row(){...}

}

Among them, the class dist_Matrix contains three attributes; dist represents the distance matrix, for example, the element dist[0][1] = 1, which represents the pairwise sequence alignment score value of the first sequence, and the second sequence is 1; row_sum represents the sum of the scores of each sequence after pairwise alignment with other sequences, that is, the row sum of dist; seqs_num represents the number of sequences participating in the alignment. In the method Dist_Matrix, seqs represents a string pointer to all sequences participating in the alignment, matrix represents a two-dimensional matrix composed of score matrix objects obtained after pairwise alignment of all sequences, and the method sum_row() is used for calculation row_sum value.

At the same time, msa_center component is transformed into a class msa_center. The attribute center_index of this class records the index of the center sequence. The method Msa_center is used to calculate the center_index, and the distance matrix object is used as its parameter. The C++ representation of this component is as follows:

class msa_center{

private:

int center_index; //record center sequence index

public:

int Msa_center(dist_Matrix distM){...}

}

rmb_space component is also converted to the class rmb_space in C++, where the attribute Msa_space_loc represents the gap position inserted when the center sequence is aligned with other sequences, and the attribute msa_ret_str means the sequence alignment result after inserting gaps in all sequences according to the “leave blank once, leave blank this time” rule. The C++ representation is as follows:

class rmb_space{

int** Msa_space_loc;//the position of the space when each sequence is aligned with the center sequence

std::string* msa_ret_str;//MAS alignment result

public:

void Msa_add_space(MsaCenterSeq mcs, Dist_Matrix distM, Msa_Sequence* seqs, Score_matrix_mani** matrix){..}

}

Through the above conversion, we can obtain the complete component library to assemble and generate the star alignment algorithm. The process of assembling and generating the star alignment algorithm is listed below, where Star represents the parameter matrix of the method Dist_Matrix used to construct the distance matrix in the star alignment algorithm, that is, the score matrix operation object in the NW algorithm.

int main{

std::string s[3]={"CGCT", "CCTG","GCGT"};

int seq_num = sizeof(s)/sizeof(s[0]);

Msa_check().check_dna(s, seq_num);

Star star(s, seq_num);

dist_Matrix distM(seq_num);

distM.Dist_Matrix(&MsaNW:Msa_NW,s, star.get_matrix());

distM.sum_row();

msa_center mc;

mc.Msa_center_seq(distM);

RmbSpace rs(seq_num, star.get_Seqs()->max_length());

rs.Msa_add_space(mc, distM, star.get_seqs(), star.get_matrix());

Msa_print_align().msa_print_align(rs.get_ret_str(), seq_num);

}

### Experiment Analysis

**Figure 2:**
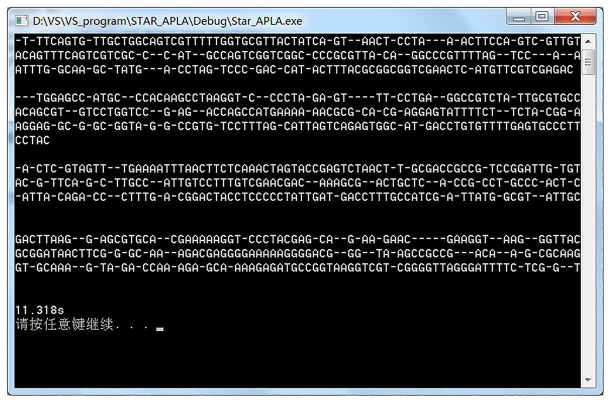
Snapshot of the alignment result.

We downloaded four *Escherichia coli* DNA data with a length of approximately 200 characters from NCBI’s Genbank gene database website for experimental testing. The basic configuration of the machine is 3.40 GHz, Intel Core i7 processor, 8 GB RAM, and Windows 7 operating system. The result of the experiment is shown in Figure 2. The comparison takes 11.318 s.

The running kr alignment algorithm generated by the assembly can perform multisequence alignment better and has obtained results similar to the original star alignment algorithm, which verifies the practicability of the star alignment algorithm generated by the assembly.

## Conclusion

Sequence alignment algorithms are widely used. Because of the complexity of multiple sequence alignment problems and the diversity of algorithm design strategies, it is difficult to guarantee the development efficiency and reliability of multiple sequence alignment algorithm programs.

This article takes the problem of multiple sequence alignment as a special field and works on the algorithm development and program generation under PAR framework. Through the analysis of problem characteristics, the generality of the domain algorithm family is extracted, the features are described, and abstract algorithm components are designed. Based on the research of the pairwise sequence alignment algorithm family, the method and platform under the PAR framework are used to assemble the specific multisequence alignment algorithms and generate programs automatically. As a case study, assembly of the star alignment algorithm is given to demonstrate the generation process of the specific algorithm program, which further proves the practicability of the component library in the related field and the reliability and efficiency of the algorithm generation under the PAR framework.

## Data Availability Statement

The datasets presented in this study can be found in online repositories. The names of the repository/repositories and accession number(s) can be found at: https://www.ncbi.nlm.nih.gov/genbank/.

## Author Contributions

HpS did the codes work and the experiments. HhS instructed the whole research work and revised the paper. SX proofread the full text. All authors read and approved the final manuscript and are agree to be accountable for all aspects of the work.

### Conflict of Interest

The authors declare that the research was conducted in the absence of any commercial or financial relationships that could be construed as a potential conflict of interest.
